# 5-(4-Fluoro­phen­yl)-2*H*-pyrazol-1-ium 2,2,2-tri­fluoro­acetate

**DOI:** 10.1107/S1600536814005200

**Published:** 2014-03-15

**Authors:** Thammarse S. Yamuna, Jerry P. Jasinski, Manpreet Kaur, Brian J. Anderson, H. S. Yathirajan

**Affiliations:** a Department of Studies in Chemistry, University of Mysore, Manasagangotri, Mysore 570 006, India; b Department of Chemistry, Keene State College, 229 Main Street, Keene, NH 03435-2001, USA

## Abstract

The title salt, C_9_H_8_FN_2_^+^·C_2_F_3_O_2_^−^, crystallizes with two independent cations (*A* and *B*) and two independent anions (*C* and *D*) in the asymmetric unit. In the cations, the dihedral angles between the benzene and pyrazolium rings are 23.7 (3)° in cation *A* and 1.8 (8)° in cation *B*. In the crystal, each anion links to the two cations *via* N—H⋯O hydrogen bonds, forming a U-shaped unit with an *R*_4_^4^(14) ring motif. These U-shaped units stack along the *a* axis and are linked *via* C—H⋯O and C—H⋯F hydrogen bonds, forming slabs lying parallel to (100). Within the slabs there are π–π inter­actions between the pyrazolium rings [inter-centroid distance = 3.6326 (15) Å] and between the benzene rings [inter-centroid distance = 3.7244 (16) Å]. In the anions, the F atoms of the tri­fluoro­methyl groups are disordered over two sets of sites, with refined occupancy ratios of 0.58 (3):0.42, 0.540 (14):0.46 (14), and 0.55 (2):0.45 (2) for anion *C*, and 0.73 (5):0.27 (5), 0.63 (5):0.37 (5), and 0.57 (8):0.43 (8) for anion *D*.

## Related literature   

For general background to pyrazole derivatives and their pharmacological activities, see: Ohno *et al.* (2004 ▶); Patel *et al.* (2010 ▶); Siu *et al.* (2008 ▶); Sullivan *et al.* (2006 ▶); Ragavan *et al.* (2009 ▶, 2010 ▶). For related structures, see: Abdul-Ghani *et al.* (1995 ▶); Ge *et al.* (2011 ▶); Han *et al.* (2011 ▶); Jasinski *et al.* (2010 ▶); Yamuna *et al.* (2013 ▶). For standard bond lengths, see: Allen *et al.* (1987 ▶).
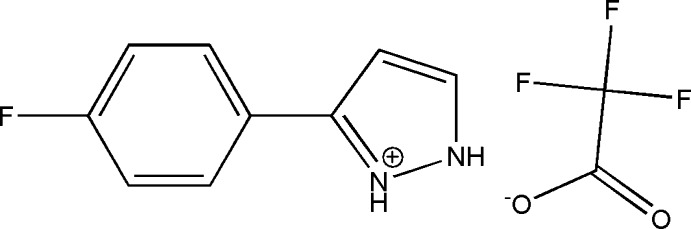


## Experimental   

### 

#### Crystal data  

C_9_H_8_FN_2_^+^·C_2_F_3_O_2_^−^
*M* *_r_* = 276.19
Monoclinic, 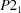

*a* = 6.7828 (2) Å
*b* = 16.8263 (6) Å
*c* = 10.4004 (4) Å
β = 93.354 (3)°
*V* = 1184.96 (8) Å^3^
*Z* = 4
Mo *K*α radiation
μ = 0.15 mm^−1^
*T* = 173 K
0.32 × 0.14 × 0.12 mm

#### Data collection  

Agilent Xcalibur (Eos, Gemini) diffractometer
Absorption correction: multi-scan (*CrysAlis PRO* and *CrysAlis RED*; Agilent, 2012 ▶) *T*_min_ = 0.867, *T*_max_ = 1.000
13825 measured reflections
7270 independent reflections
5352 reflections with *I* > 2σ(*I*)
*R*_int_ = 0.029

#### Refinement  

*R*[*F* ^2^ > 2σ(*F* ^2^)] = 0.049
*wR*(*F* ^2^) = 0.110
*S* = 1.02
7270 reflections
419 parameters
1 restraint
H atoms treated by a mixture of independent and constrained refinement
Δρ_max_ = 0.18 e Å^−3^
Δρ_min_ = −0.21 e Å^−3^

### 

Data collection: *CrysAlis PRO* (Agilent, 2012 ▶); cell refinement: *CrysAlis PRO*; data reduction: *CrysAlis RED* (Agilent, 2012 ▶); program(s) used to solve structure: *SUPERFLIP* (Palatinus & Chapuis, 2007 ▶); program(s) used to refine structure: *SHELXL2012* (Sheldrick, 2008 ▶); molecular graphics: *OLEX2* (Dolomanov *et al.*, 2009 ▶); software used to prepare material for publication: *OLEX2*.

## Supplementary Material

Crystal structure: contains datablock(s) I. DOI: 10.1107/S1600536814005200/su2707sup1.cif

Structure factors: contains datablock(s) I. DOI: 10.1107/S1600536814005200/su2707Isup2.hkl

Supporting information file. DOI: 10.1107/S1600536814005200/su2707Isup3.cml

CCDC reference: 990481

Additional supporting information:  crystallographic information; 3D view; checkCIF report

## Figures and Tables

**Table 1:** Hydrogen-bond geometry (Å, °)

*D*—H⋯*A*	*D*—H	H⋯*A*	*D*⋯*A*	*D*—H⋯*A*
N1*B*—H1*B*⋯O2*D*	0.94 (4)	1.75 (4)	2.656 (3)	162 (4)
N2*B*—H2*B*⋯O2*C*	0.87 (5)	1.77 (5)	2.634 (3)	175 (4)
N1*A*—H1*A*⋯O1*D* ^i^	0.98 (4)	1.69 (4)	2.665 (3)	170 (3)
N2*A*—H2*A*⋯O1*C* ^i^	0.88 (4)	1.77 (4)	2.649 (3)	180 (4)
C1*B*—H1*BA*⋯O1*C* ^ii^	0.93	2.59	3.256 (3)	129
C2*B*—H2*BA*⋯O1*D* ^ii^	0.93	2.48	3.384 (3)	165
C5*B*—H5*B*⋯O2*C*	0.93	2.50	3.385 (4)	159
C9*A*—H9*A*⋯F2*DA* ^iii^	0.93	2.39	3.26 (3)	156

Symmetry codes: (i) 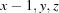; (ii) 
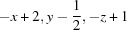
; (iii) 
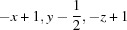
.

**Fig. 1.**
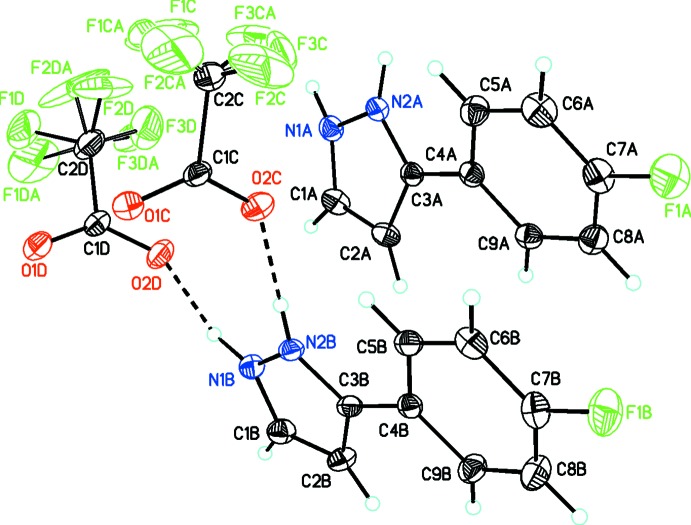
A view of the molecular structure of the title salt, with atom labelling. Displacement ellipsoids are drawn at the 30% probability level.

**Fig. 2.**
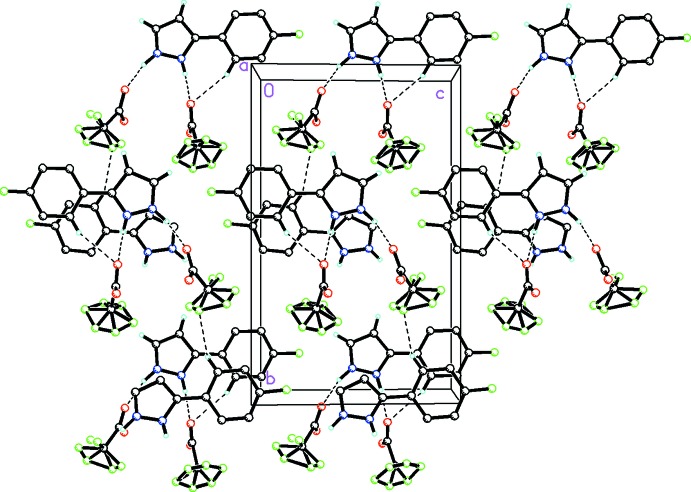
A view along the a axis of the crystal packing of the title compound. The N—H···O, C-H···F and C-H···O hydrogen bonds are shown as dashed lines (see Table 1 for details; H atoms not involved in hydrogen bonding have been omitted for clarity).

## supplementary crystallographic information

### 1. Comment

Pyrazoles and their derivatives exhibit a variety of pharmacological properties, for example, antibacterial and anti-inflammatory activities (Sullivan *et al.*, 2006; Patel *et al.*, 2010), nucleosidase inhibitory activity against staphylococcusaureus (Siu *et al.*, 2008), and antimicrobial activity (Ragavan *et al.* 2009, 2010). Fluorinated pyrazoles have also been shown to possess interesting biological activities, for example, as herbicides (Ohno *et al.*, 2004). Recently, crystal structures of 3,5-bis(4-fluorophenyl)-1-phenyl-4,5-dihydro-1H-pyrazole (Jasinski *et al.*, 2010), 3-aminopyrazolium trifluoroacetate (Yamuna *et al.*, 2013) have been reported by our research group. The crystal structures of some related compounds, viz., 1-trifluoroacetyl-3-trifluoromethyl-3a,8b-dihydro-1H,4H-indeno[1,2-c]pyrazole (Abdul-Ghani *et al.*, 1995), ethyl 1-(4-chlorobenzyl)-3-(4-fluorophenyl)-1H-pyrazole-5-carboxylate (Ge *et al.*, 2011) and ethyl 1-benzyl-3-(4-fluorophenyl)-1H-pyrazole-5-carboxylate (Han *et al.*, 2011) have been reported. In view of the importance of pyrazole derivatives, herein we report on the crystal structure of the title salt.

The title salt crystallizes with two independent cations (A and B) and two independent anions (C and D) in the asymmetric unit (Fig 1). In the cations, the dihedral angles between the benzene and pyrazolium rings is 23.7 (3)° in cation A and 1.8 (8)° in cation B. The bond lengths are in normal ranges (Allen *et al.*, 1987).

In the crystal, each anion links to the two cations via N-H···O hydrogen bonds forming a U-shaped unit with an R^4^_4_(14) ring motif (Table 1 and Fig. 2). These U-shaped units stack along the a axis and are linked via C-H···O and C-H···F hydrogen bonds forming slabs lying parallel to (100) [Fig. 2 and Table 1]. Within the slabs there are π—π interactions involving the pyrazolium rings (Cg1—Cg3^i^ = 3.6326 (15) Å) and between the benzene rings (Cg2—Cg4^i^ = 3.7244 (16) Å) [where Cg1, Cg2, Cg3 and Cg4 are the centroids of rings N1A/N2A/C1A-C3A, C4A–C9A, N1B/N2B/C1B-C3B and C4B–C9B, respectively; symmetry code: (i) x-1, y, z].

### 2. Experimental

3-(4-Fluoro-phenyl)-1H-pyrazole (0.2 g, 3.0833 mmol; Sigma-Aldrich) was dissolved in a mixture of trifluoroacetic and methanol (1:3 v/v) and stirred for 10 minutes at 313 K. The resulting solution was allowed to cool slowly at room temperature, yielding colourless block-like crystals of the title compound after a few days (M.p: 353-358 K).

### 3. Refinement

The NH H atoms were located in a difference Fourier map and freely refined. The C-bound H atoms were placed in calculated positions and refined using a riding model: C-H = 0.93 and 0.97Å for CH and CH_2_ H atoms, respectively, with U_iso_(H) = 1.2*U*_eq_(C). In the anions, disorder was modeled for the fluorine atoms of the trifluoromethyl groups over two sets of sites with occupancy ratios of 0.58 (3):0.42 (F1C), 0.540 (14):0.46 (14) (F2C), 0.55 (2):0.45 (2) (F3C) and 0.73 (5):0.27 (5) (F1D), 0.63 (5):0.37 (5) (F2D), and 0.57 (8):0.43 (8) (F3D).

### Crystal data

**Table:**

C_9_H_8_FN_2_^+^·C_2_F_3_O_2_^−^	*F*(000) = 560
*M**_r_* = 276.19	*D*_x_ = 1.548 Mg m^−^^3^
Monoclinic, *P*2_1_	Mo *K*α radiation, λ = 0.71073 Å
*a* = 6.7828 (2) Å	Cell parameters from 3244 reflections
*b* = 16.8263 (6) Å	θ = 3.0–32.8°
*c* = 10.4004 (4) Å	µ = 0.15 mm^−^^1^
β = 93.354 (3)°	*T* = 173 K
*V* = 1184.96 (8) Å^3^	Block, colourless
*Z* = 4	0.32 × 0.14 × 0.12 mm

### Data collection

**Table:**

Agilent Xcalibur (Eos, Gemini) diffractometer	7270 independent reflections
Radiation source: Enhance (Mo) X-ray Source	5352 reflections with *I* > 2σ(*I*)
Detector resolution: 16.0416 pixels mm^-1^	*R*_int_ = 0.029
ω scans	θ_max_ = 32.9°, θ_min_ = 3.0°
Absorption correction: multi-scan (*CrysAlis PRO* and *CrysAlis RED*; Agilent, 2012)	*h* = −9→9
*T*_min_ = 0.867, *T*_max_ = 1.000	*k* = −24→23
13825 measured reflections	*l* = −15→15

### Refinement

**Table:**

Refinement on *F*^2^	Primary atom site location: structure-invariant direct methods
Least-squares matrix: full	Hydrogen site location: mixed
*R*[*F*^2^ > 2σ(*F*^2^)] = 0.049	H atoms treated by a mixture of independent and constrained refinement
*wR*(*F*^2^) = 0.110	*w* = 1/[σ^2^(*F*_o_^2^) + (0.0401*P*)^2^ + 0.1594*P*] where *P* = (*F*_o_^2^ + 2*F*_c_^2^)/3
*S* = 1.02	(Δ/σ)_max_ < 0.001
7270 reflections	Δρ_max_ = 0.18 e Å^−^^3^
419 parameters	Δρ_min_ = −0.21 e Å^−^^3^
1 restraint	

### Special details

**Table:**

Geometry. All esds (except the esd in the dihedral angle between two l.s. planes) are estimated using the full covariance matrix. The cell esds are taken into account individually in the estimation of esds in distances, angles and torsion angles; correlations between esds in cell parameters are only used when they are defined by crystal symmetry. An approximate (isotropic) treatment of cell esds is used for estimating esds involving l.s. planes.

### Fractional atomic coordinates and isotropic or equivalent isotropic displacement parameters (Å^2^)

**Table:**

	*x*	*y*	*z*	*U*_iso_*/*U*_eq_	Occ. (<1)
F1D	0.7862 (10)	0.7079 (9)	0.8704 (15)	0.089 (4)	0.73 (5)
F1DA	0.793 (4)	0.668 (4)	0.912 (3)	0.123 (11)	0.27 (5)
F2D	0.6499 (18)	0.7180 (7)	0.6830 (13)	0.112 (4)	0.63 (5)
F2DA	0.686 (5)	0.7292 (9)	0.731 (5)	0.152 (11)	0.37 (5)
F3D	0.558 (2)	0.6299 (6)	0.823 (2)	0.081 (4)	0.57 (8)
F3DA	0.543 (3)	0.6355 (16)	0.788 (5)	0.114 (7)	0.43 (8)
O1D	1.0279 (3)	0.63369 (12)	0.7099 (2)	0.0408 (5)	
O2D	0.7848 (3)	0.54440 (13)	0.6797 (2)	0.0499 (6)	
C1D	0.8581 (4)	0.60784 (15)	0.7155 (3)	0.0335 (5)	
C2D	0.7130 (5)	0.6654 (2)	0.7773 (4)	0.0560 (9)	
F1C	0.7172 (16)	0.7924 (4)	0.330 (2)	0.144 (9)	0.58 (3)
F1CA	0.743 (2)	0.7792 (12)	0.237 (2)	0.121 (8)	0.42 (3)
F2C	0.4749 (11)	0.7172 (5)	0.3343 (11)	0.095 (5)	0.540 (14)
F2CA	0.624 (3)	0.7661 (11)	0.4070 (14)	0.179 (9)	0.460 (14)
F3C	0.628 (2)	0.7258 (9)	0.1734 (6)	0.116 (5)	0.55 (2)
F3CA	0.503 (3)	0.7082 (5)	0.248 (3)	0.214 (16)	0.45 (2)
O1C	0.9686 (3)	0.67332 (13)	0.3635 (2)	0.0491 (5)	
O2C	0.7104 (3)	0.59074 (12)	0.3406 (2)	0.0547 (6)	
C1C	0.7934 (5)	0.65565 (15)	0.3406 (3)	0.0365 (6)	
C2C	0.6556 (6)	0.72546 (19)	0.3014 (4)	0.0599 (10)	
F1B	0.6835 (3)	0.37120 (13)	−0.20823 (17)	0.0577 (5)	
N1B	0.8500 (3)	0.42473 (13)	0.5212 (2)	0.0329 (5)	
H1B	0.844 (5)	0.461 (2)	0.589 (4)	0.058 (10)*	
N2B	0.8131 (3)	0.44327 (13)	0.3969 (2)	0.0284 (4)	
H2B	0.783 (7)	0.492 (3)	0.375 (4)	0.077 (14)*	
C1B	0.8796 (4)	0.34634 (17)	0.5313 (3)	0.0372 (6)	
H1BA	0.9083	0.3185	0.6073	0.045*	
C2B	0.8602 (4)	0.31413 (16)	0.4097 (3)	0.0349 (6)	
H2BA	0.8738	0.2609	0.3881	0.042*	
C3B	0.8161 (3)	0.37683 (15)	0.3249 (2)	0.0266 (5)	
C4B	0.7787 (3)	0.37670 (16)	0.1853 (2)	0.0270 (5)	
C5B	0.7429 (3)	0.44680 (16)	0.1155 (3)	0.0309 (5)	
H5B	0.7399	0.4951	0.1587	0.037*	
C6B	0.7119 (4)	0.44506 (18)	−0.0168 (3)	0.0360 (6)	
H6B	0.6890	0.4916	−0.0635	0.043*	
C7B	0.7158 (4)	0.3729 (2)	−0.0777 (3)	0.0391 (6)	
C8B	0.7496 (5)	0.30331 (18)	−0.0135 (3)	0.0443 (7)	
H8B	0.7517	0.2554	−0.0581	0.053*	
C9B	0.7808 (4)	0.30496 (16)	0.1193 (3)	0.0364 (6)	
H9B	0.8032	0.2578	0.1645	0.044*	
F1A	0.1746 (3)	0.46388 (14)	−0.16103 (17)	0.0633 (6)	
N1A	0.2469 (3)	0.54317 (14)	0.5635 (2)	0.0342 (5)	
H1A	0.179 (5)	0.578 (2)	0.622 (3)	0.054 (10)*	
N2A	0.2056 (3)	0.55446 (14)	0.4364 (2)	0.0304 (4)	
H2A	0.127 (5)	0.594 (2)	0.412 (3)	0.045 (9)*	
C1A	0.3425 (4)	0.47499 (18)	0.5801 (3)	0.0374 (6)	
H1AA	0.3878	0.4537	0.6589	0.045*	
C2A	0.3641 (4)	0.44055 (17)	0.4604 (3)	0.0334 (5)	
H2AA	0.4243	0.3923	0.4433	0.040*	
C3A	0.2767 (3)	0.49337 (14)	0.3714 (2)	0.0272 (5)	
C4A	0.2539 (3)	0.48770 (15)	0.2306 (2)	0.0287 (5)	
C5A	0.2251 (4)	0.55489 (17)	0.1529 (3)	0.0375 (6)	
H5A	0.2229	0.6051	0.1902	0.045*	
C6A	0.1999 (5)	0.5467 (2)	0.0206 (3)	0.0444 (7)	
H6A	0.1812	0.5911	−0.0320	0.053*	
C7A	0.2032 (4)	0.47215 (19)	−0.0312 (3)	0.0410 (7)	
C8A	0.2336 (4)	0.40478 (18)	0.0413 (3)	0.0379 (6)	
H8A	0.2373	0.3550	0.0028	0.045*	
C9A	0.2588 (4)	0.41308 (16)	0.1737 (3)	0.0305 (5)	
H9A	0.2792	0.3682	0.2250	0.037*	

### Atomic displacement parameters (Å^2^)

**Table:**

	*U*^11^	*U*^22^	*U*^33^	*U*^12^	*U*^13^	*U*^23^
F1D	0.075 (4)	0.083 (6)	0.109 (6)	−0.001 (3)	0.018 (3)	−0.066 (5)
F1DA	0.160 (17)	0.13 (3)	0.076 (13)	0.036 (13)	0.030 (9)	−0.068 (16)
F2D	0.148 (6)	0.081 (6)	0.106 (6)	0.084 (6)	0.007 (6)	0.008 (4)
F2DA	0.215 (19)	0.022 (5)	0.23 (2)	0.023 (8)	0.147 (18)	0.008 (9)
F3D	0.083 (7)	0.041 (5)	0.126 (7)	−0.002 (4)	0.070 (6)	−0.009 (6)
F3DA	0.043 (6)	0.118 (14)	0.184 (17)	−0.015 (6)	0.031 (8)	−0.081 (11)
O1D	0.0453 (11)	0.0305 (10)	0.0475 (12)	−0.0042 (9)	0.0114 (9)	−0.0045 (8)
O2D	0.0491 (12)	0.0356 (11)	0.0655 (15)	−0.0043 (9)	0.0070 (10)	−0.0206 (10)
C1D	0.0442 (15)	0.0257 (12)	0.0309 (13)	0.0004 (10)	0.0038 (11)	−0.0035 (10)
C2D	0.052 (2)	0.0357 (17)	0.082 (3)	−0.0051 (14)	0.0195 (18)	−0.0178 (17)
F1C	0.095 (7)	0.019 (3)	0.30 (2)	0.009 (3)	−0.123 (10)	−0.021 (6)
F1CA	0.131 (9)	0.084 (10)	0.146 (15)	0.002 (7)	−0.019 (9)	0.085 (10)
F2C	0.053 (4)	0.064 (5)	0.171 (10)	0.032 (4)	0.036 (6)	0.030 (5)
F2CA	0.23 (2)	0.111 (11)	0.197 (12)	0.118 (12)	−0.002 (11)	−0.057 (9)
F3C	0.133 (9)	0.148 (10)	0.064 (4)	0.069 (8)	−0.023 (4)	0.032 (4)
F3CA	0.142 (14)	0.033 (4)	0.44 (4)	−0.005 (7)	−0.221 (19)	0.004 (11)
O1C	0.0460 (12)	0.0312 (11)	0.0684 (15)	0.0051 (9)	−0.0115 (10)	−0.0022 (9)
O2C	0.0573 (14)	0.0239 (11)	0.0811 (17)	0.0021 (9)	−0.0101 (12)	0.0003 (10)
C1C	0.0483 (16)	0.0226 (12)	0.0370 (14)	0.0065 (11)	−0.0096 (12)	−0.0027 (10)
C2C	0.058 (2)	0.0259 (15)	0.092 (3)	0.0019 (14)	−0.028 (2)	0.0049 (16)
F1B	0.0747 (14)	0.0629 (12)	0.0345 (9)	−0.0106 (11)	−0.0057 (9)	−0.0004 (9)
N1B	0.0306 (11)	0.0331 (12)	0.0351 (13)	0.0018 (8)	0.0032 (9)	−0.0047 (9)
N2B	0.0262 (10)	0.0213 (10)	0.0378 (12)	0.0013 (8)	0.0036 (8)	−0.0006 (8)
C1B	0.0400 (15)	0.0345 (14)	0.0371 (15)	0.0060 (11)	0.0019 (12)	0.0019 (11)
C2B	0.0432 (15)	0.0234 (12)	0.0382 (15)	0.0047 (11)	0.0043 (11)	−0.0005 (10)
C3B	0.0228 (10)	0.0219 (10)	0.0357 (13)	0.0012 (9)	0.0056 (9)	0.0003 (10)
C4B	0.0209 (10)	0.0265 (11)	0.0339 (12)	−0.0020 (9)	0.0033 (9)	0.0002 (10)
C5B	0.0256 (11)	0.0259 (12)	0.0414 (15)	−0.0006 (9)	0.0040 (10)	0.0013 (10)
C6B	0.0297 (12)	0.0397 (15)	0.0385 (15)	−0.0012 (11)	0.0013 (11)	0.0099 (12)
C7B	0.0376 (14)	0.0469 (16)	0.0323 (14)	−0.0073 (13)	−0.0018 (11)	0.0001 (12)
C8B	0.057 (2)	0.0367 (16)	0.0384 (16)	−0.0037 (13)	−0.0010 (13)	−0.0072 (12)
C9B	0.0437 (15)	0.0268 (13)	0.0383 (15)	−0.0012 (11)	−0.0005 (11)	−0.0014 (11)
F1A	0.0772 (14)	0.0833 (16)	0.0299 (10)	0.0056 (12)	0.0085 (9)	−0.0018 (9)
N1A	0.0292 (11)	0.0404 (13)	0.0329 (12)	0.0009 (9)	0.0015 (9)	−0.0032 (9)
N2A	0.0259 (10)	0.0298 (11)	0.0351 (12)	−0.0004 (8)	−0.0022 (8)	−0.0005 (9)
C1A	0.0323 (13)	0.0458 (17)	0.0345 (14)	0.0048 (11)	0.0035 (11)	0.0057 (12)
C2A	0.0287 (12)	0.0380 (14)	0.0341 (14)	0.0049 (10)	0.0056 (10)	0.0066 (11)
C3A	0.0213 (10)	0.0277 (12)	0.0326 (13)	−0.0024 (9)	0.0018 (9)	0.0012 (9)
C4A	0.0227 (11)	0.0315 (13)	0.0319 (13)	−0.0015 (9)	0.0029 (9)	0.0016 (10)
C5A	0.0430 (15)	0.0320 (14)	0.0375 (15)	−0.0048 (11)	0.0015 (12)	0.0031 (11)
C6A	0.0466 (16)	0.0470 (17)	0.0398 (17)	−0.0019 (14)	0.0046 (13)	0.0131 (13)
C7A	0.0358 (14)	0.059 (2)	0.0292 (14)	0.0001 (13)	0.0064 (11)	−0.0013 (13)
C8A	0.0323 (13)	0.0418 (15)	0.0405 (16)	−0.0011 (11)	0.0100 (12)	−0.0076 (12)
C9A	0.0249 (11)	0.0323 (13)	0.0348 (14)	0.0000 (9)	0.0056 (10)	0.0020 (10)

### Geometric parameters (Å, º)

**Table:**

F1D—C2D	1.281 (8)	C4B—C9B	1.389 (4)
F1DA—C2D	1.47 (3)	C5B—H5B	0.9300
F2D—C2D	1.371 (9)	C5B—C6B	1.380 (4)
F2DA—C2D	1.19 (2)	C6B—H6B	0.9300
F3D—C2D	1.324 (11)	C6B—C7B	1.370 (4)
F3DA—C2D	1.270 (18)	C7B—C8B	1.361 (4)
O1D—C1D	1.236 (3)	C8B—H8B	0.9300
O2D—C1D	1.226 (3)	C8B—C9B	1.385 (4)
C1D—C2D	1.546 (4)	C9B—H9B	0.9300
F1C—C2C	1.232 (6)	F1A—C7A	1.360 (3)
F1CA—C2C	1.291 (11)	N1A—H1A	0.98 (4)
F2C—C2C	1.299 (7)	N1A—N2A	1.349 (3)
F2CA—C2C	1.322 (11)	N1A—C1A	1.324 (4)
F3C—C2C	1.333 (8)	N2A—H2A	0.88 (4)
F3CA—C2C	1.183 (8)	N2A—C3A	1.336 (3)
O1C—C1C	1.234 (4)	C1A—H1AA	0.9300
O2C—C1C	1.229 (3)	C1A—C2A	1.389 (4)
C1C—C2C	1.541 (4)	C2A—H2AA	0.9300
F1B—C7B	1.363 (3)	C2A—C3A	1.390 (4)
N1B—H1B	0.94 (4)	C3A—C4A	1.467 (3)
N1B—N2B	1.340 (3)	C4A—C5A	1.397 (4)
N1B—C1B	1.337 (3)	C4A—C9A	1.389 (4)
N2B—H2B	0.87 (5)	C5A—H5A	0.9300
N2B—C3B	1.346 (3)	C5A—C6A	1.384 (4)
C1B—H1BA	0.9300	C6A—H6A	0.9300
C1B—C2B	1.375 (4)	C6A—C7A	1.366 (4)
C2B—H2BA	0.9300	C7A—C8A	1.371 (4)
C2B—C3B	1.396 (4)	C8A—H8A	0.9300
C3B—C4B	1.459 (3)	C8A—C9A	1.384 (4)
C4B—C5B	1.399 (4)	C9A—H9A	0.9300
			
O1D—C1D—C2D	114.6 (2)	C4B—C5B—H5B	119.6
O2D—C1D—O1D	130.8 (3)	C6B—C5B—C4B	120.8 (3)
O2D—C1D—C2D	114.5 (3)	C6B—C5B—H5B	119.6
F1D—C2D—F2D	105.7 (11)	C5B—C6B—H6B	120.9
F1D—C2D—F3D	105.0 (11)	C7B—C6B—C5B	118.3 (3)
F1D—C2D—C1D	115.9 (5)	C7B—C6B—H6B	120.9
F1DA—C2D—C1D	101.8 (14)	F1B—C7B—C6B	118.2 (3)
F2D—C2D—C1D	106.7 (6)	C8B—C7B—F1B	118.9 (3)
F2DA—C2D—F1DA	114 (4)	C8B—C7B—C6B	122.9 (3)
F2DA—C2D—F3DA	106 (3)	C7B—C8B—H8B	120.6
F2DA—C2D—C1D	119.1 (13)	C7B—C8B—C9B	118.9 (3)
F3D—C2D—F2D	109.2 (10)	C9B—C8B—H8B	120.6
F3D—C2D—C1D	114.0 (6)	C4B—C9B—H9B	119.8
F3DA—C2D—F1DA	103 (3)	C8B—C9B—C4B	120.3 (3)
F3DA—C2D—C1D	113.0 (9)	C8B—C9B—H9B	119.8
O1C—C1C—C2C	115.4 (2)	N2A—N1A—H1A	117 (2)
O2C—C1C—O1C	130.5 (3)	C1A—N1A—H1A	133 (2)
O2C—C1C—C2C	114.0 (3)	C1A—N1A—N2A	108.7 (2)
F1C—C2C—F2C	110.2 (10)	N1A—N2A—H2A	118 (2)
F1C—C2C—F3C	105.3 (12)	C3A—N2A—N1A	109.1 (2)
F1C—C2C—C1C	116.2 (4)	C3A—N2A—H2A	132 (2)
F1CA—C2C—F2CA	99.9 (15)	N1A—C1A—H1AA	125.6
F1CA—C2C—C1C	112.3 (6)	N1A—C1A—C2A	108.7 (2)
F2C—C2C—F3C	100.6 (7)	C2A—C1A—H1AA	125.6
F2C—C2C—C1C	114.6 (5)	C1A—C2A—H2AA	127.3
F2CA—C2C—C1C	107.5 (6)	C1A—C2A—C3A	105.5 (2)
F3C—C2C—C1C	108.3 (4)	C3A—C2A—H2AA	127.3
F3CA—C2C—F1CA	110.5 (16)	N2A—C3A—C2A	107.9 (2)
F3CA—C2C—F2CA	109.4 (17)	N2A—C3A—C4A	122.3 (2)
F3CA—C2C—C1C	116.0 (5)	C2A—C3A—C4A	129.7 (2)
N2B—N1B—H1B	124 (2)	C5A—C4A—C3A	121.9 (2)
C1B—N1B—H1B	127 (2)	C9A—C4A—C3A	118.7 (2)
C1B—N1B—N2B	108.9 (2)	C9A—C4A—C5A	119.4 (2)
N1B—N2B—H2B	120 (3)	C4A—C5A—H5A	120.0
N1B—N2B—C3B	109.6 (2)	C6A—C5A—C4A	120.0 (3)
C3B—N2B—H2B	130 (3)	C6A—C5A—H5A	120.0
N1B—C1B—H1BA	125.9	C5A—C6A—H6A	120.7
N1B—C1B—C2B	108.1 (3)	C7A—C6A—C5A	118.7 (3)
C2B—C1B—H1BA	125.9	C7A—C6A—H6A	120.7
C1B—C2B—H2BA	126.6	F1A—C7A—C6A	118.8 (3)
C1B—C2B—C3B	106.8 (2)	F1A—C7A—C8A	118.0 (3)
C3B—C2B—H2BA	126.6	C6A—C7A—C8A	123.2 (3)
N2B—C3B—C2B	106.6 (2)	C7A—C8A—H8A	121.0
N2B—C3B—C4B	123.2 (2)	C7A—C8A—C9A	118.0 (3)
C2B—C3B—C4B	130.2 (2)	C9A—C8A—H8A	121.0
C5B—C4B—C3B	122.0 (2)	C4A—C9A—H9A	119.7
C9B—C4B—C3B	119.1 (2)	C8A—C9A—C4A	120.7 (2)
C9B—C4B—C5B	118.9 (2)	C8A—C9A—H9A	119.7
			
O1D—C1D—C2D—F1D	−35.8 (11)	C1B—C2B—C3B—C4B	−179.3 (2)
O1D—C1D—C2D—F1DA	−67 (3)	C2B—C3B—C4B—C5B	−177.8 (2)
O1D—C1D—C2D—F2D	81.5 (8)	C2B—C3B—C4B—C9B	1.4 (4)
O1D—C1D—C2D—F2DA	58 (3)	C3B—C4B—C5B—C6B	178.7 (2)
O1D—C1D—C2D—F3D	−157.9 (12)	C3B—C4B—C9B—C8B	−178.7 (2)
O1D—C1D—C2D—F3DA	−177 (3)	C4B—C5B—C6B—C7B	0.5 (4)
O2D—C1D—C2D—F1D	144.7 (11)	C5B—C4B—C9B—C8B	0.6 (4)
O2D—C1D—C2D—F1DA	113 (3)	C5B—C6B—C7B—F1B	179.5 (2)
O2D—C1D—C2D—F2D	−98.0 (8)	C5B—C6B—C7B—C8B	−0.3 (4)
O2D—C1D—C2D—F2DA	−121 (3)	C6B—C7B—C8B—C9B	0.3 (5)
O2D—C1D—C2D—F3D	22.6 (12)	C7B—C8B—C9B—C4B	−0.4 (4)
O2D—C1D—C2D—F3DA	4 (3)	C9B—C4B—C5B—C6B	−0.6 (3)
O1C—C1C—C2C—F1C	−19.2 (15)	F1A—C7A—C8A—C9A	−178.7 (2)
O1C—C1C—C2C—F1CA	33.1 (15)	N1A—N2A—C3A—C2A	0.9 (3)
O1C—C1C—C2C—F2C	−149.6 (7)	N1A—N2A—C3A—C4A	179.4 (2)
O1C—C1C—C2C—F2CA	−75.8 (14)	N1A—C1A—C2A—C3A	0.7 (3)
O1C—C1C—C2C—F3C	99.0 (9)	N2A—N1A—C1A—C2A	−0.1 (3)
O1C—C1C—C2C—F3CA	161 (2)	N2A—C3A—C4A—C5A	24.1 (4)
O2C—C1C—C2C—F1C	162.5 (15)	N2A—C3A—C4A—C9A	−155.1 (2)
O2C—C1C—C2C—F1CA	−145.2 (14)	C1A—N1A—N2A—C3A	−0.5 (3)
O2C—C1C—C2C—F2C	32.1 (8)	C1A—C2A—C3A—N2A	−0.9 (3)
O2C—C1C—C2C—F2CA	105.9 (13)	C1A—C2A—C3A—C4A	−179.3 (2)
O2C—C1C—C2C—F3C	−79.3 (9)	C2A—C3A—C4A—C5A	−157.8 (3)
O2C—C1C—C2C—F3CA	−17 (2)	C2A—C3A—C4A—C9A	23.0 (4)
F1B—C7B—C8B—C9B	−179.5 (3)	C3A—C4A—C5A—C6A	−178.6 (2)
N1B—N2B—C3B—C2B	−0.9 (3)	C3A—C4A—C9A—C8A	178.6 (2)
N1B—N2B—C3B—C4B	179.2 (2)	C4A—C5A—C6A—C7A	0.2 (4)
N1B—C1B—C2B—C3B	−0.3 (3)	C5A—C4A—C9A—C8A	−0.6 (4)
N2B—N1B—C1B—C2B	−0.2 (3)	C5A—C6A—C7A—F1A	178.7 (3)
N2B—C3B—C4B—C5B	2.1 (3)	C5A—C6A—C7A—C8A	−1.1 (5)
N2B—C3B—C4B—C9B	−178.6 (2)	C6A—C7A—C8A—C9A	1.1 (4)
C1B—N1B—N2B—C3B	0.7 (3)	C7A—C8A—C9A—C4A	−0.2 (4)
C1B—C2B—C3B—N2B	0.7 (3)	C9A—C4A—C5A—C6A	0.6 (4)

### Hydrogen-bond geometry (Å, º)

**Table:**

*D*—H···*A*	*D*—H	H···*A*	*D*···*A*	*D*—H···*A*
N1*B*—H1*B*···O2*D*	0.94 (4)	1.75 (4)	2.656 (3)	162 (4)
N2*B*—H2*B*···O2*C*	0.87 (5)	1.77 (5)	2.634 (3)	175 (4)
N1*A*—H1*A*···O1*D*^i^	0.98 (4)	1.69 (4)	2.665 (3)	170 (3)
N2*A*—H2*A*···O1*C*^i^	0.88 (4)	1.77 (4)	2.649 (3)	180 (4)
C1*B*—H1*BA*···O1*C*^ii^	0.93	2.59	3.256 (3)	129
C2*B*—H2*BA*···O1*D*^ii^	0.93	2.48	3.384 (3)	165
C5*B*—H5*B*···O2*C*	0.93	2.50	3.385 (4)	159
C9*A*—H9*A*···F2*DA*^iii^	0.93	2.39	3.26 (3)	156

Symmetry codes: (i) *x*−1, *y*, *z*; (ii) −*x*+2, *y*−1/2, −*z*+1; (iii) −*x*+1, *y*−1/2, −*z*+1.
